# The Clinical Utility of Informants' Appraisals on Prospective and Retrospective Memory in Patients with Early Alzheimer's Disease

**DOI:** 10.1371/journal.pone.0112210

**Published:** 2014-11-10

**Authors:** Yen-Hsuan Hsu, Ching-Feng Huang, Min-Chien Tu, Mau-Sun Hua

**Affiliations:** 1 Department of Psychology, College of Science, National Taiwan University, Taipei, Taiwan; 2 Department of Neurology, Taichung Tzu Chi Hospital, Buddhist Tzu Chi Medical Foundation, Taichung, Taiwan; 3 Departments of Psychiatry and Neurology, National Taiwan University Hospital, College of Medicine, National Taiwan University, Taipei, Taiwan; 4 Graduate Institute of Brain and Mind Sciences, College of Medicine, National Taiwan University, Taipei, Taiwan; 5 Neurobiological and Cognitive Science Center, National Taiwan University, Taipei, Taiwan; University of California, San Francisco, United States of America

## Abstract

Increasing studies suggest the importance of including prospective memory measures in clinical evaluation of dementia due to its sensitivity and functional relevance. The Prospective and Retrospective Memory Questionnaire (PRQM) is originally a self-rated memory inventory that offers a direct comparison between prospective and episodic memory. However, the informant's report has been recognized as a more valid source of cognitive complaints. We thus aimed to examine the validity of the informant-rated form of the PRMQ in assessing memory function of the patients and in detecting individuals with early dementia. The informants of 140 neurological outpatients with memory complaints completed the Taiwan version of the PRMQ. Tests of prospective memory, short-term memory, and general cognitive ability were also administered to non-demented participants and patients with early stages of Alzheimer's disease (AD). Results showed significant relationships between the PRMQ ratings and objective cognitive measures, and showed that higher ratings on the PRMQ were associated with increasing odds of greater dementia severity. Receiver operative characteristic (ROC) curves showed an adequate ability of the PRMQ to identify patients with dementia (93% sensitivity and 84% specificity). Hierarchical regression revealed that the PRMQ has additional explanatory power for dementia status after controlling for age, education and objective memory test results, and that the prospective memory subscale owns predictive value for dementia beyond the retrospective memory subscale. The present study demonstrated the external validity and diagnostic value of informants' evaluation of their respective patients' prospective and retrospective memory functioning, and highlighted the important role of prospective memory in early dementia detection. The proxy-version of the PRMQ is a useful tool that captures prospective and episodic memory problems in patients with early AD, in combination with standardized cognitive testing.

## Introduction

Alzheimer's disease (AD) is the leading cause of dementia syndromes. Early detection is an important task, considering the nature of progressive course and possible disease-modifying therapies [1], [2]. As neurofibrillary tangles and senile plaques gradually spread from mesial temporal structures to the isocortex [3], memory impairment is often manifested as the first symptom [4]. In addition to traditionally assessed episodic memory, accumulating studies suggest the importance of incorporating the assessment of prospective memory into neuropsychological evaluation, given that it has been proven to be sensitive to the early stages of AD [5]–[9] and mild cognitive impairment [10]–[15]. Moreover, studies indicate that prospective memory shows greater functional implications than episodic memory for activities of daily living [16] and healthcare [16]–[18].

Prospective memory refers to the memory of an action to be performed at a future time [19], [20]. It is a psychological construct that correlates to but is dissociable from episodic memory [21]–[23]. It is comprised of multi-componential processes that require an individual to realize an intended action is to be performed when the proper cue is encountered (i.e., the prospective component), and to spontaneously recall the intention after the cue is detected (i.e., the retrospective component) [24]. Prospective memory is believed to be related to episodic memory as well as to attention and executive function [25], [26], and relies on the synergistic functioning of a distributed neural network, including the temporal and the prefrontal areas [27], [28]. Researchers have argued that involvement of the frontal and cingulate areas in the early dementing process may exacerbate the functional change in mesial temporal areas and are thus responsible for early impairment of prospective memory [12], [15], [29].

Nonetheless, assessment of prospective memory is time-consuming, preventing it from being adopted into clinical routine. Therefore, development of a more efficient tool would be beneficial. The Prospective and Retrospective Memory Questionnaire (PRMQ) [30] is a brief self-reported memory questionnaire that evaluates prospective and retrospective memory, and offers a direct comparison between the two. The PRMQ has been used in several studies to measure subjective memory complaints or to reflect subjects' everyday memory functions [31]–[35], and has been proven psychometrically sound [36]–[39]. Although a questionnaire does not equate to objective test results, it provides information about real-life situations and outperforms performance-based tests in terms of time and supervision.

However, the source of reports may have a great impact on the reliability of a questionnaire. Self-reported data of the patients with AD who manifest anosognosia or decreased awareness of their memory loss [40], [41] may be inaccurate. Furthermore, studies showed that informant-corroborated report of memory impairment is superior to self-reported results in predicting future diagnosis of dementia [42], [43]. Crawford, Henry, Ward, and Blake [44] examined the psychometric properties of the proxy-rated version of the PRMQ in a healthy population and showed acceptable reliability and construct validity. There has been no further study investigating the clinical utility of proxy-rated version of the PRMQ. Thus, we have chosen to investigate the feasibility of applying the informant-rated PRMQ to patients with subjective memory functioning concerns in clinical practice.

The objective of the present study was to examine the clinical utility of the informant's report of the patient's memory impairments on the PRMQ, given its convenience, time-saving, and real-life relevance. We aimed to confirm four assumptions: First, there are correlations between informant-ratings on the PRMQ and objective cognitive measures. Second, there are differences between informants' ratings for patients at different stages of dementia. Third, the informant-rated PRMQ yields acceptable sensitivity and specificity in detecting dementia, as reflected by Receiver Operative (ROC) curves. Fourth, there is an incremental explanatory power of the informant-rated PRMQ for detecting dementia after controlling for age, education, and objective memory test.

## Participants and Methods

### Participants

One hundred and forty neurological outpatients and their informants who completed the PRMQ were enrolled in the present study according to inclusion and exclusion criteria. These patients visited the memory clinic of the department of neurology at a regional general hospital in Taiwan from June to December 2013. Patients with memory complaints who received a cognitive evaluation were included. Exclusion criteria included: age younger than 45; history of stroke; head injury with loss of consciousness for more than 30 minutes; neurological diseases; and psychiatric disorders (e.g., individuals diagnosed with Major Depressive Disorder).

Information from clinical interviews performed by neurologists and clinical neuropsychologists, cognitive tests, laboratory results, and brain imaging findings (28 patients had magnetic resonance imaging and 75 patients had computerized tomography results) was collected. Medical history was obtained by family interviews. Participants were classified into three stages according to the Clinical Dementia Rating Scale (CDR) [45]: no dementia (CDR = 0); questionable or very mild dementia (CDR = 0.5); and mild dementia (CDR = 1). Among these patients, 21 were classified as having no dementia (CDR = 0); 59 were classified as having questionable dementia (CDR = 0.5); and 60 were classified as belonging to the mild dementia group (CDR = 1). The diagnosis of probable AD was made according to the National Institute of Neurological and Communicative Disorders and Stroke (NINCDS) and the Alzheimer's Disease and Related Disorders Association (ADRDA) [4] under consensus of a neurologist and a clinical neuropsychologist. In total, 90 patients met the criteria of probable AD. Twelve patients were found to have a single earlier infarction; none had multiple infarctions or confluent white matter changes. Among all the participants, 66% of them had hypertension and 36% of them had diabetes mellitus all under medication control. The study was approved by the Institutional Research Board (IRB) of Taichung Tzu Chi Hospital. Written informed consent was approved to be waived by the IRB. Raw data are not publicized considering the potential violation of confidentiality. However, data underlying the findings are available upon request to the corresponding author.

### Materials

All patients received the Mini-Mental Status Examination (MMSE) [46] and their informants completed the Prospective and Retrospective Memory Questionnaire (PRMQ) [47] that assesses the frequency of everyday memory performance failures. Items are rated on a 5-point Likert-type scale ranging from 1 (never) to 5 (very often). The Taiwan version of the PRMQ [37] has a 5-item prospective memory scale (e.g., Do you forget appointments if you are not prompted by someone else or by a reminder such as a calendar or diary?) and a 6-item retrospective memory scale (e.g., Do you fail to recall things that have happened to you in the last few days?). It has demonstrated acceptable internal consistency (Cronbach's alpha  = 0.82–0.90), as well as a tripartite structure with one general episodic memory factor and one specific prospective memory factor. The patients also received the Cognitive Ability Screening Instrument (CASI) [48] and two single-trial prospective memory tasks. In the current study, we focused on the short-term memory index of the CASI as a measure of episodic memory, which includes two trials of delayed verbal memory and an object recognition test.

The single-trial prospective memory tasks included an event-based task and a time-based task. Each task was scored by a combination of the prospective component score and the retrospective component score. The former assessed whether the participant remembered that an action was to be accomplished and the latter assessed the content of the action. The Envelope Test is an event-based prospective memory task adapted from previous studies [6], [9], [49]. Participants were told that at a later time the examiner would give them an envelope to hold. When this happened, they were required to remember to seal the envelope with a round sticker of a specific color (counterbalanced between red and blue). After a 10-minute delay, the envelope was presented. For the prospective component score, the patient had to remember and intend to do something after receiving the envelope. The patient was given 2 points if something was done within 15 seconds after the envelope was given (even if the action was incorrect), 1 point if it was done late; and 0 points if no intention was shown within 30 seconds. If no action was performed within 30 seconds, a prompt would be given in order to assess the retrospective component. For the retrospective component score, the patient received 2 points if the envelope was sealed with the correct color either with or without a prompt, 1 point if another color was used, and 0 if the wrong action or no action was carried out. The score ranged from 0 to 4 in this task.

The Telephone Test is a time-based prospective memory measure we modified from the prompt card task [9], [49]. The participants were requested to remind the examiner to make a phone call to the counter 5 minutes after the instruction was given. A prompt was given if no action was performed within 1 minute of the proper time. For the prospective component score, a score of 2 was given if the patient spontaneously reminded the examiner that something needed to be done within 1 minute of the correct timing, 1 point if the reminder was given earlier or later than 1 minute, and 0 if the patient did not act. For the retrospective component score, the patient received 2 points if the action was remembered correctly; 1 point if the patient did not remember the instruction precisely but remembered it was something to do with the counter or telephone; and 0 points if no instruction was remembered or the content was irrelevant. Scores ranged from 0 to 4 on this task.

### Statistical Analysis

Demographic and cognitive variables of each group were illustrated by descriptive statistics and compared by analysis of variance (ANOVA). Wilcoxon signed-rank tests were employed to examine the difference between mean ratings of the prospective and retrospective memory subscales within each severity group. Zero-order correlations were performed by Spearman sign-rank correlations to examine the relationships between age, education, cognitive performance, and ratings on questionnaires. Cronbach's *α* to test the reliability of the scales was also calculated.

We conducted multinomial logistic regression to examine dementia severity as a function of the PRMQ ratings, while covarying for age and education. We coded our dependent variable (i.e., dementia severity) as an ordinal outcome (1 for CDR = 0; 2 for CDR = 0.5; and 3 for CDR = 1). In this multinomial logistic regression model, exponentiated coefficients are calculated as ratios of relative probabilities, thus can be called conditional odds ratios (COR).

Receiver operating characteristic (ROC) curves were created to test the clinical utility of informant-reported PRMQ, and to set the cut-off score. Optimum cut-off scores for the PRMQ were determined at the maximum Youden index (J =  sensitivity + specificity −1) level [50]. The area under the curves (AUCs) was examined to show sensitivity and specificity of the test variables for identifying dementia. It is generally accepted that an AUC value greater than 0.9 indicates high accuracy, 0.7–0.9 indicates moderate accuracy, and 0.5–0.6 indicates low accuracy [50].

The hierarchical regression model was used to examine the incremental power of the PRMQ ratings after accounting for the influence of demographic variables and objective short-term memory measure. The incremental power of the prospective memory subscale was also examined, controlling for the influence of age and the retrospective memory subscale. Many of these variables had skewed distributions; however, the distributions of the regression residuals were normally distributed, and this analytic approach was deemed appropriate. All statistical tests were performed by using SPSS version 19. Values are presented as mean and standard deviation (*SD*), and *p*<0.05 indicated statistical significance.

## Results

Demographic data and the summary of measurement results are shown in Table 1. There were significant group differences of age (*F*_2, 137_ = 26.46; *p*<0.001) and education (*F*_2, 137_ = 3.17; *p* = 0.05) between the groups. Wilcoxon signed-rank tests revealed significantly higher mean ratings for the prospective memory subscale than for the retrospective memory subscale in each group, but to a greater extent in the very mild dementia group (CDR = 0.5; *Z* = −5.27; *p*<0.001) as compared with the no-dementia group (CDR = 0; *Z* = −2.56; *p* = 0.01) and the mild dementia group (CDR = 1; *Z* = −2.13; *p* = 0.03).

**Table 1:** Demographics, informant ratings on PRMQ, and cognitive measures.

	CDR = 0		CDR = 0.5		CDR = 1	
	Mean	*SD*	Mean	*SD*	Mean	*SD*
Age^‡^	65.14	8.65	72.02	8.79	79.25	7.33
Education^*^	7.86	4.20	5.39	3.93	5.40	4.30
MMSE^‡^	26.43	2.52	23.03	3.90	17.37	4.86
Short-term memory index^‡^	7.24	2.91	5.15	3.18	2.50	1.73
Prospective memory tasks^‡^	5.54	1.81	2.42	1.97	0.84	1.44
PRMQ-PM^‡^	9.86	2.57	16.54	4.30	21.43	3.59
PRMQ-RM^‡^	10.10	3.48	17.31	5.24	25.02	4.38
PRMQ-total^‡^	19.95	5.31	33.85	9.10	46.45	7.64

*Notes*: CDR: Clinical Dementia Rating Scale; PRMQ: Prospective and Retrospective Memory Questionnaire; PM: Prospective Memory subscale; RM: Retrospective memory subscale; MMSE: Mini-Mental Status Examination; *p<0.05; ^‡^ *p<0.001*.

As can be seen in Table 2, Spearman correlations revealed significant relationships of the PRMQ ratings with age (*ρ* = 0.30 ∼ 0.38; *p*<0.001), but not with education (*ρ* = −0.08∼−0.12; p = 0.16∼0.37). Significant associations were also shown between the PRMQ ratings and the MMSE (*N* = 140; *ρ* = −0.45∼−0.53; *p*<0.001), short-term memory (*N* = 140; *ρ* = −0.44∼−0.46; *p*<0.001), and prospective memory tasks (*N* = 88; *ρ* = −0.57∼−0.58; *p*<0.001). Cronbach's *α* was 0.97 for the total scale, 0.94 for the prospective memory subscale, and 0.93 for the retrospective memory subscale, yielding appropriate internal consistency. Table 3 shows the association of the PRMQ ratings with the odds of dementia severity, covarying for age and education. In addition to age, ratings on the PRMQ were a significant predictor in models, indicating that a higher PRMQ rating was associated with higher odds of greater dementia severity (COR = 1.30 and COR = 1.55 for patients with CDR 0.5 and CDR 1, respectively).

**Table 2:** Spearman correlations of informant-rated PRMQ, demographics, and cognitive measures.

	PRMQ-PM	PRMQ-RM	PRMQ-total score
Age	0.30^‡^	0.38^‡^	0.35^‡^
Education	−0.08	−0.12	−0.10
MMSE	−0.45^‡^	−0.53^‡^	−0.51^‡^
Short-term memory	−0.44^‡^	−0.46^‡^	−0.46^‡^
Prospective memory	−0.57^‡^	−0.58^‡^	−0.58^‡^

*Notes*: PRMQ: Prospective and Retrospective Memory Questionnaire; PM: Prospective Memory subscale; RM: Retrospective memory subscale; MMSE: Mini-Mental Status Examination; ^‡^ *p*<0.001.

**Table 3:** Multinomial logistic regression of dementia severity on PRMQ, with age and education as covariates, and stratified by subscales.

	Conditional odds ratio (COR)	Standard error	*p*	95% Confidence interval
**PRMQ total scale**				
CDR 0 vs. 0.5				
Age	1.09	0.04	0.039	1.01–1.19
Education	0.96	0.09	0.669	0.82–1.14
PRMQ	1.30	0.07	<0.001	1.14–1.49
CDR 0 vs. 1				
Age	1.26	0.06	<0.001	1.13–1.40
Education	1.02	0.11	0.854	0.83–1.25
PRMQ	1.55	0.08	<0.001	1.33–1.80
**Prospective Memory subscale (PRMQ-PM)**				
CDR 0 vs. 0.5				
Age	1.10	0.04	0.023	1.01–1.20
Education	0.97	0.09	0.704	0.81–1.15
PRMQ-PM	1.71	0.14	<0.001	1.31–2.22
CDR 0 vs. 1				
Age	1.28	0.06	<0.001	1.15–1.42
Education	1.01	0.11	0.912	0.82–1.24
PRMQ-PM	2.37	0.15	<0.001	1.76–3.19
**Retrospective Memory subscale (PRMQ-RM)**				
CDR 0 vs. 0.5				
Age	1.07	0.04	0.078	0.99–1.15
Education	0.94	0.09	0.487	0.80–1.12
PRMQ-RM	1.46	0.10	<0.001	1.20–1.78
CDR 0 vs. 1				
Age	1.22	0.05	<0.001	1.10–1.35
Education	1.00	0.11	0.997	0.81–1.23
PRMQ-RM	1.97	0.12	<0.001	1.57–2.48

**Figure 1:**
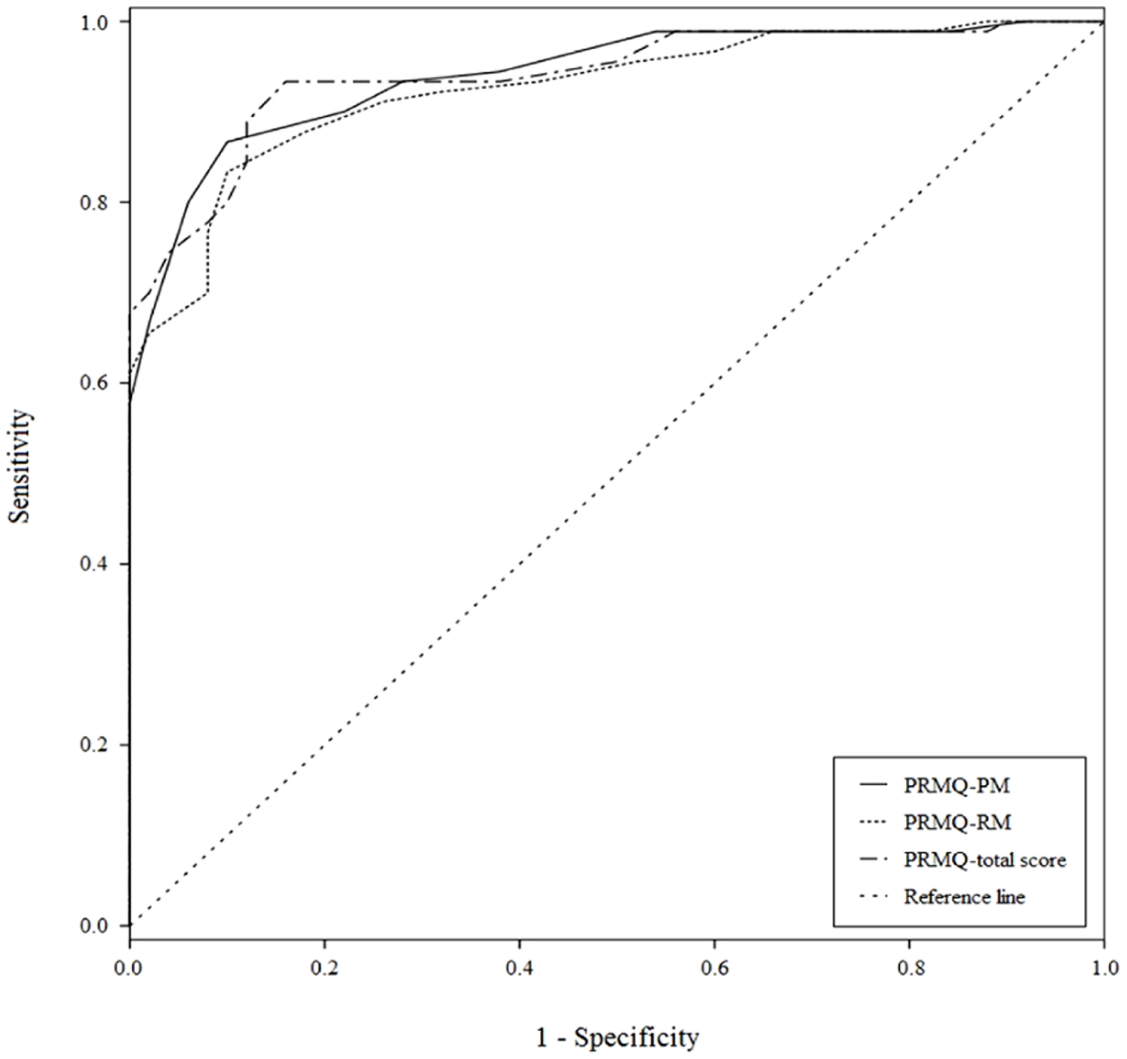
Receiver-operating characteristic (ROC) curves. The ROC curves for the Prospective and Retrospective Memory Questionnaire (PRMQ) ratings as screening tests for dementia. *Notes*: PRMQ-PM: the prospective memory subscale of PRMQ; PRMQ-RM: the retrospective memory subscale of PRMQ.

By using ROC analysis for the prospective memory subscale, retrospective memory subscale, and the PRMQ total scale, the calculated AUC values were 0.94, 0.93, and 0.94, respectively (Table 4 and Figure 1). Therefore, our analysis indicated that the PRMQ and its subscales had acceptable accuracy in identifying dementia. Based on the maximum Youden index, the optimum cut-off point was 31.5 (mean rating of 2.86 point) for the PRMQ total score, with 93% sensitivity and 84% specificity; 16.5 (mean rating of 3.3 points) for the prospective memory subscale with 87% sensitivity and 90% specificity; and 18.5 (mean rating of 3.08) for the retrospective memory subscale with 83% sensitivity and 90% specificity.

**Table 4:** Sensitivity and specificity of PRMQ using optimal cut-off scores.

	AUC	Cut-off	Sensitivity	Specificity
PRMQ-PM	0.94	16.5	87%	90%
PRMQ-RM	0.93	18.5	83%	90%
PRMQ-total score	0.94	31.5	93%	84%

*Notes*: PRMQ: Prospective and Retrospective Memory Questionnaire; PM: Prospective memory subscale; RM: Retrospective memory subscale; AUC: Area under the curve.

Hierarchical linear regression conducted to predict dementia status (see Table 5) showed an incremental explanatory power of informant-rated PRMQ after controlling for age, education, and objective short-term memory measure. The inclusion of PRMQ variables in the second step of the model significantly increased in the proportion of dementia status variance explained (*p*<0.001; *Δ*R^2^ = 0.327). In addition, when controlling for age and ratings on the retrospective memory subscale (Table 6), ratings on the prospective memory subscale still showed incremental power (*p*<0.001; *Δ*R^2^ = 0.058).

**Table 5:** Hierarchical regression concurrently predicting dementia from PRMQ after controlling for age, education and traditional memory test.

Predictors	*B*	*β*	R^2^	Adj. R^2^	*Δ*R^2^
**Step 1**			0.278	0.262^‡^	
Age	0.014	0.279^†^			
Education	−0.002	−0.021			
Short-term memory	−0.047	−0.307^†^			
**Step 2**			0.604	0.593^‡^	0.327^‡^
Age	0.009	0.178^†^			
Education	−0.010	−0.089			
Short-term memory	−0.003	−0.020			
PRMQ	0.026	0.661^‡^			

*Notes*: B: Beta weight; *β*: standardized beta weight; Adj.: adjusted; *Δ*: change; PRMQ: Prospective and Retrospective Memory Questionnaire; ^†^ *p*<0.01; ^‡^ *p*<0.001.

**Table 6:** Hierarchical regression concurrently predicting dementia from the prospective memory subscale after controlling for age, education, and retrospective memory subscale.

Predictors	*B*	*β*	R^2^	Adj. R^2^	*Δ*R^2^
**Step 1**			0.560	0.551^‡^	
Age	0.009	0.180^†^			
Education	−0.010	−0.091			
PRMQ-RM	0.043	0.636^‡^			
**Step 2**			0.618	0.607^‡^	0.058^‡^
Age	0.011	0.209^†^			
Education	−0.011	−0.098			
PRMQ-RM	0.007	0.103			
PRMQ-PM	0.050	0.574^‡^			

*Notes*: B: Beta weight; *β*: standardized beta weight; Adj.: adjusted; *Δ*: change; PRMQ: Prospective and Retrospective Memory Questionnaire; PRMQ-RM: the prospective memory subscale of the PRMQ; PRMQ-PM: the retrospective memory subscale of the PRMQ; ^†^ *p*<0.01; ^‡^ *p*<0.001.

## Discussion

The current study aimed to examine the validity and diagnostic value of the informant-rated PRMQ in clinical settings. Results confirmed our previous assumptions. First, the informants' ratings on the PRMQ were significantly associated with objective measures of short-term memory, prospective memory, and general cognitive ability. Second, higher frequency of memory failures, as reflected by the PRMQ, was associated with higher odds of greater dementia severity. Third, the informants' ratings on the PRMQ displayed good sensitivity and specificity in identifying dementia patients. Fourth, the informant-rated PRMQ demonstrated incremental validity over demographics and objective memory test in detecting dementia. Our findings confirmed that informants' ratings of patients' prospective and retrospective memory problems can accurately reflect their impairments, and supported the utility of the informant's evaluation on the PRMQ as an alternative memory measure for clinical practice [44].

Our study supported the view that prospective memory measure is sensitive to the early stages of dementia [6] and that the performance of prospective memory made an independent contribution to the prediction of AD beyond that of retrospective memory performance [7]. The high frequency of prospective memory may be secondary to the impairment of retrospective memory; however, the additional deficits of other cognitive elements during the progressive course of Alzheimer's disease may exacerbate prospective memory problems. In addition, neuroimaging studies [51]–[54] have observed decreased functional connectivity and myelin breakdown of the associated cortical regions in the early stages of AD. The patients may thus show vulnerability to cognitive processes that require coordination of a distributed neural network, such as a prospective memory task. Alternatively, it is possible that prospective rather than retrospective memory failures induced a greater life impact more noticeable to the informants [30], [31], rendering an incremental power of detecting dementia status. Overall, our results highlighted the value of informants' appraisals on the patients' prospective memory problems in early dementia detection.

There have been several well-established memory questionnaires that assess the patients' memory function or the informants' report of the patients' function in real life, such as the Everyday Memory Questionnaire (EMQ) [55], [56], the Memory Assessment Clinics-Self Rating Scale (MAC-S) [57] and the Short Memory Questionnaire (SMQ) [58]. However, prior questionnaires did not adequately assess prospective memory because they sampled larger constructs. The current study is the first to demonstrate that the informant-rated version of the PRMQ is a reliable and valid questionnaire featuring additional and specific assessment of prospective memory in patients with early dementia.

Some proxy-rated questionnaires have been shown to be effective in detecting patients with dementia or mild cognitive impairment, such as the AD8 [59] and the Informant Questionnaire on Cognitive Decline in the Elderly (IQCODE) [60]. These diagnostic tools usually contain items describing multiple cognitive domains and functional status and ask informants to rate the patient's current status as compared to their premorbid status. The informant-rated PRMQ did not outperform these well-validated tools in detecting dementia or patients with mild cognitive impairment [61], [62]. However, our major interest was to explore whether the informant's quantitative ratings on the patient's everyday memory problems may provide valid information about the patient's objective ability and thus supplement clinical judgment.

An informant-rated memory questionnaire is a reliable dementia-screening tool with several merits in clinical settings. First, studies consistently showed correlations between the objective test performance and the informant's reports of memory [42], [63], and showed that the informant's observation of memory decline increases diagnostic accuracy for dementia [43], [64]. Second, informant-reported memory impairment is often the reason of referral to a memory clinic, and thus, it is a clinical routine to clarify the nature of these memory complaints. Third, informant reports are less vulnerable to patient's uncooperativeness or physical disabilities than taking cognitive tests. Fourth, some neuropsychological tests bear limited ecologic validity [65], whereas the informant's observation ought to provide information about the patient's everyday memory function. A thorough understanding of the patient's real-life memory problems may also be of help with the future planning of rehabilitation. Other minor merits, such as requiring less professional training, minimal cost, attainability and so forth, also promote the value of this screening method.

There are several limitations in the current study. First, the educational level of our sample was relatively low. This may compromise the representativeness and limit the generalization of the current results. However, analysis showed that there is little influence of patients' educational level on the informant's ratings of the memory questionnaire. In fact, the high occurrence of illiterate patients in our hospital has restricted the use of several neuropsychological test tools. One of the advantages of the PRMQ is that the patient's prior academic attainment is not requisite. Second, the items covered in the prospective memory subscale describe more of the general rather than specific performance of the patient's prospective memory in daily life, and provide little information about which process problem is responsible for performance failure. The subscale thus merely provides an overall index serving as a screening tool for further assessment. Third, patients in the non-dementia group were those with subjective memory complaints but showed normal cognitive function on objective cognitive testing. These patients may be in an early stage of memory deterioration. However, our study demonstrated that informant-rated PRMQ is capable of reflecting the patient's memory impairment and may be used to indicate dementia status.

In sum, the current study demonstrated the external validity and diagnostic value of informants' evaluation of their respective patients' prospective and retrospective memory functioning, and highlighted the important role of prospective memory in early dementia detection. The proxy-version of the PRMQ is a useful tool that captures prospective and episodic memory problems in patients with early AD, in collaboration with standardized cognitive testing.

## References

[1] Nestor PJ, Scheltens P, Hodges JR (2004) Advances in the early detection of Alzheimer's disease. Nat Med 10: S34–S41. 15298007 10.1038/nrn1433

[2] Petersen RC, Doody R, Kurz A, Mohs RC, Morris JC, et al (2001) Current concepts in mild cognitive impairment. Arch Neurol 58: 1985–1992. 11735772 10.1001/archneur.58.12.1985

[3] Braak H, Braak E (1991) Neuropathological staging of Alzheimer-related changes. Acta Neuropathol 82: 239–259. 1759558 10.1007/BF00308809

[4] McKhann GM, Knopman DS, Chertkow H, Hyman BT, Jack CR, et al (2011) The diagnosis of dementia due to Alzheimer's disease: Recommendations from the National Institute on Aging-Alzheimer's Association workgroups on diagnostic guidelines for Alzheimer's disease. Alzheimers Dement 7: 263–269. 21514250 10.1016/j.jalz.2011.03.005 PMC3312024

[5] Duchek JM, Balota DA, Cortese M (2006) Prospective memory and apolipoprotein E in healthy aging and early stage Alzheimer's disease. Neuropsychology 20: 633–644. 17100508 10.1037/0894-4105.20.6.633

[6] Huppert FA, Beardsall L (1993) Prospective memory impairment as an early indicator of dementia. J Clin Exp Neuropsychol 15: 805–821. 8276937 10.1080/01688639308402597

[7] Jones S, Livner A, Backman L (2006) Patterns of prospective and retrospective memory impairment in preclinical Alzheimer's disease. Neuropsychology 20: 144–152. 16594775 10.1037/0894-4105.20.2.144

[8] Martins SP, Damasceno BP (2008) Prospective and retrospective memory in mild Alzheimer's disease. Arq Neuropsiquiatr 66: 318–322. 18641863 10.1590/s0004-282x2008000300006

[9] Kinsella G, Ong B, Storey E, Wallace J, Hester R (2007) Elaborated spaced-retrieval and prospective memory in mild Alzheimer's disease. Neuropsychol Rehabil 17: 688–706. 17852763 10.1080/09602010600892824

[10] Blanco-Campal A, Coen RF, Lawlor BA, Walsh JB, Burke TE (2009) Detection of prospective memory deficits in mild cognitive impairment of suspected Alzheimer's disease etiology using a novel event-based prospective memory task. J Int Neuropsychol Soc 15: 154–159. 19128540 10.1017/S1355617708090127

[11] Costa A, Perri R, Serra L, Barban F, Gatto I, et al (2010) Prospective memory functioning in mild cognitive impairment. Neuropsychology 24: 327–335. 20438210 10.1037/a0018015

[12] Karantzoulis S, Troyer AK, Rich JB (2009) Prospective memory in amnestic mild cognitive impairment. J Int Neuropsychol Soc 15: 407–415. 19402927 10.1017/S1355617709090596

[13] Kazui H, Matsuda A, Hirono N, Mori E, Miyoshi N, et al (2005) Everyday Memory Impairment of Patients with Mild Cognitive Impairment. Dement Geriatr Cogn Disord 19: 331–337. 15785034 10.1159/000084559

[14] Thompson C, Henry JD, Rendell P, Withall A, Brodaty H (2010) Prospective memory function in mild cognitive impairment and early dementia. J Int Neuropsychol Soc 16: 318–325. 20128933 10.1017/S1355617709991354

[15] Troyer AK, Murphy KJ (2007) Memory for intentions in amnestic mild cognitive impairment: time- and event-based prospective memory. J Int Neuropsychol Soc 13: 365–369. 17286894 10.1017/S1355617707070452

[16] Woods SP, Weinborn M, Velnoweth A, Rooney A, Bucks RS (2011) Memory for Intentions is Uniquely Associated with Instrumental Activities of Daily Living in Healthy Older Adults. J Int Neuropsychol Soc: 1–5. 10.1017/S1355617711001263 PMC3268683 22032776

[17] Woods SP, Dawson MS, Weber E, Gibson S, Grant I, et al (2009) Timing is everything: antiretroviral nonadherence is associated with impairment in time-based prospective memory. J Int Neuropsychol Soc 15: 42–52. 19128527 10.1017/S1355617708090012 PMC2776623

[18] Dieckmann P, Reddersen S, Wehner T, Rall M (2006) Prospective memory failures as an unexplored threat to patient safety: results from a pilot study using patient simulators to investigate the missed execution of intentions. Ergonomics 49: 526–543. 16717009 10.1080/00140130600568782

[19] Kvavilashvili L (1998) Remembering intentions: testing a new method of investigation. Appl Cogn Psychol 12: 533–554.

[20] McDaniel MA, Einstein GO (2000) Strategic and automatic processes in prospective memory retrieval: A multiprocess framework. Appl Cogn Psychol 14: S127–S144.

[21] Cockburn J (1995) Task interruption in prospective memory: a frontal lobe function? Cortex 31: 87–97. 7781322 10.1016/s0010-9452(13)80107-4

[22] Zeintl M, Kliegel M, Hofer SA (2007) The role of processing resources in age-related prospective and retrospective memory within old age. Psychology and Aging 22: 826–834. 18179300 10.1037/0882-7974.22.4.826

[23] Salthouse TA, Berish DE, Siedlecki KL (2004) Construct validity and age sensitivity of prospective memory. Mem Cognit 32: 1133–1148. 10.3758/bf03196887 15813495

[24] Dobbs AR, Rule BG (1987) Prospective Memory and Self-Reports of Memory Abilities in Older Adults. Can J Exp Psychol 41: 209–222. 10.1037/h0084152 3502897

[25] Clune-Ryberg M, Blanco-Campal A, Carton S, Pender N, O′Brien D, et al (2011) The contribution of retrospective memory, attention and executive functions to the prospective and retrospective components of prospective memory following TBI. Brain Inj 25: 819–831. 21721845 10.3109/02699052.2011.589790

[26] Schnitzspahn KM, Stahl C, Zeintl M, Kaller CP, Kliegel M (2012) The Role of Shifting, Updating, and Inhibition in Prospective Memory Performance in Young and Older Adults. Dev Psychol 49: 1544–1553. 23148933 10.1037/a0030579

[27] Simons JS, Scholvinck ML, Gilbert SJ, Frith CD, Burgess PW (2006) Differential components of prospective memory? Evidence from fMRI. Neuropsychologia 44: 1388–1397. 16513147 10.1016/j.neuropsychologia.2006.01.005

[28] West R, Krompinger J (2005) Neural correlates of prospective and retrospective memory. Neuropsychologia 43: 418–433. 15707617 10.1016/j.neuropsychologia.2004.06.012

[29] Burgess PW, Quayle A, Frith CD (2001) Brain regions involved in prospective memory as determined by positron emission tomography. Neuropsychologia 39: 545–555. 11257280 10.1016/s0028-3932(00)00149-4

[30] Smith G, Della Sala S, Logie RH, Maylor EA (2000) Prospective and retrospective memory in normal ageing and dementia: a questionnaire study. Memory 8: 311–321. 11045239 10.1080/09658210050117735

[31] Woods SP, Weinborn M, Velnoweth A, Rooney A, Bucks RS (2012) Memory for intentions is uniquely associated with instrumental activities of daily living in healthy older adults. J Int Neuropsychol Soc 18: 134–138. 22032776 10.1017/S1355617711001263 PMC3268683

[32] Eschen A, Martin M, Gasser US, Kliegel M (2009) Prospective and retrospective memory complaints in mild cognitive impairment and mild Alzheimer's disease. Brain Impairment 10: 59–75.

[33] Singer JJ, Falchi M, MacGregor AJ, Cherkas LF, Spector TD (2006) Genome-wide scan for prospective memory suggests linkage to chromosome 12q22. Behav Genet 36: 18–28. 16378169 10.1007/s10519-005-9011-1

[34] Heffernan TM, O′Neill TS, Moss M (2012) Smoking-related prospective memory deficits in a real-world task. Drug Alcohol Depend 120: 1–6. 21726964 10.1016/j.drugalcdep.2011.06.010

[35] Zeintl M, Kliegel M, Rast P, Zimprich D (2006) Prospective memory complaints can be predicted by prospective memory performance in older adults. Dement Geriatr Cogn Disord 22: 209–215. 16899998 10.1159/000094915

[36] Ronnlund M, Vestergren P, Mantyla T, Nilsson LG (2011) Predictors of self-reported prospective and retrospective memory in a population-based sample of older adults. J Genet Psychol 172: 266–284. 21902005 10.1080/00221325.2010.538450

[37] Hsu YH, Hua MS (2011) Taiwan Version of the Prospective and Retrospective Memory Questionnaire: Latent Structure and Normative Data. Arch Clin Neuropsychol 26: 240–249. 21421567 10.1093/arclin/acr012

[38] Piauilino DC, Bueno OFA, Tufik S, Bittencourt LR, Santos-Silva R, et al (2010) The Prospective and Retrospective Memory Questionnaire: A population-based random sampling study. Memory 18: 413–426. 20408038 10.1080/09658211003742672

[39] Crawford J, Smith G, Maylor E, Della Sala S, Logie R (2003) The Prospective and Retrospective Memory Questionnaire (PRMQ): Normative data and latent structure in a large non-clinical sample. Memory 11: 261–275. 12908675 10.1080/09658210244000027

[40] Cosentino S, Metcalfe J, Butterfield B, Stern Y (2007) Objective Metamemory Testing Captures Awareness of Deficit in Alzheimer's Disease. Cortex 43: 1004–1019. 17941356 10.1016/s0010-9452(08)70697-x PMC2676685

[41] Roberts JL, Clare L, Woods RT (2009) Subjective Memory Complaints and Awareness of Memory Functioning in Mild Cognitive Impairment: A Systematic Review. Dement Geriatr Cogn Disord 28: 95–109. 19684399 10.1159/000234911

[42] Carr DB, Gray S, Baty J, Morris JC (2000) The value of informant versus individual's complaints of memory impairment in early dementia. Neurology 55: 1724–1726. 11113230 10.1212/wnl.55.11.1724

[43] Rabin LA, Wang CL, Katz MJ, Derby CA, Buschke H, et al (2012) Predicting Alzheimer's Disease: Neuropsychological Tests, Self-Reports, and Informant Reports of Cognitive Difficulties. J Am Geriatr Soc 60: 1128–1134. 22690986 10.1111/j.1532-5415.2012.03956.x PMC3375855

[44] Crawford JR, Henry JD, Ward AL, Blake J (2006) The Prospective and Retrospective Memory Questionnaire (PRMQ): Latent structure, normative data and discrepancy analyses for proxy-ratings. Br J Clin Psychol 45: 83–104. 16480568 10.1348/014466505X28748

[45] Morris JC (1993) The Clinical Dementia Rating (CDR) - current version and scoring rules. Neurology 43: 2412–2414. 10.1212/wnl.43.11.2412-a 8232972

[46] Folstein MF, Folstein SE, McHugh PR (1975) “Mini-mental state”. A practical method for grading the cognitive state of patients for the clinician. J Psychiatr Res 12: 189–198. 1202204 10.1016/0022-3956(75)90026-6

[47] Smith G, Petersen RC, Ivnik RJ, Malec JF, Tangalos EG (1996) Subjective memory complaints, psychological distress, and longitudinal change in objective memory performance. Psychol Aging 11: 272–279. 8795055 10.1037//0882-7974.11.2.272

[48] Lin KN, Wang PN, Liu HC, Teng EL (2012) Cognitive Abilities Screening Instrument, Chinese Version 2.0 (CASI C-2.0): administration and clinical application. Acta Neurol Taiwan 21: 180–189. 23329550

[49] Delprado J, Kinsella G, Ong B, Pike K, Ames D, et al (2012) Clinical Measures of Prospective Memory in Amnestic Mild Cognitive Impairment. J Int Neuropsychol Soc 18: 295–304. 22264396 10.1017/S135561771100172X

[50] Akobeng AK (2007) Understanding diagnostic tests 3: Receiver operating characteristic curves. Acta Paediatr 96: 644–647. 17376185 10.1111/j.1651-2227.2006.00178.x

[51] Zhuang L, Sachdev PS, Trollor JN, Kochan NA, Reppermund S, et al (2012) Microstructural white matter changes in cognitively normal individuals at risk of amnestic MCI. Neurology 79: 748–754. 22843270 10.1212/WNL.0b013e3182661f4d

[52] Bartzokis G (2004) Age-related myelin breakdown: a developmental model of cognitive decline and Alzheimer's disease. Neurobiol Aging 25: 5–18 author reply 49–62. 14675724 10.1016/j.neurobiolaging.2003.03.001

[53] Agosta F, Pievani M, Sala S, Geroldi C, Galluzzi S, et al (2011) White matter damage in Alzheimer disease and its relationship to gray matter atrophy. Radiology 258: 853–863. 21177393 10.1148/radiol.10101284

[54] Grady CL, Furey ML, Pietrini P, Horwitz B, Rapoport SI (2001) Altered brain functional connectivity and impaired short-term memory in Alzheimer's disease. Brain 124: 739–756. 11287374 10.1093/brain/124.4.739

[55] Sunderland A, Harris JE, Baddeley AD (1983) Do laboratory tests predict everyday memory? A neuropsychological study. J Verb Learn Verb Beh 22: 341–357.

[56] Cornish IM (2000) Factor structure of the everyday memory questionnaire. Br J Psychol 91: 427–438. 10958583 10.1348/000712600161916

[57] Crook TH 3rd, Larrabee GJ (1990) A self-rating scale for evaluating memory in everyday life. Psychol Aging 5: 48–57. 2317301 10.1037//0882-7974.5.1.48

[58] Koss E, Patterson MB, Ownby R, Stuckey JC, Whitehouse PJ (1993) Memory evaluation in Alzheimer's disease. Caregivers' appraisals and objective testing. Arch Neurol 50: 92–97. 8418807 10.1001/archneur.1993.00540010086023

[59] Galvin JE, Roe CM, Powlishta KK, Coats MA, Muich SJ, et al (2005) The AD8: a brief informant interview to detect dementia. Neurology 65: 559–564. 16116116 10.1212/01.wnl.0000172958.95282.2a

[60] Jorm AF, Jacomb PA (1989) The Informant Questionnaire on Cognitive Decline in the Elderly (IQCODE): socio-demographic correlates, reliability, validity and some norms. Psychol Med 19: 1015–1022. 2594878 10.1017/s0033291700005742

[61] Dong Y, Pang WS, Lim LB, Yang YH, Morris JC, et al (2013) The informant AD8 is superior to participant AD8 in detecting cognitive impairment in a memory clinic setting. J Alzheimers Dis 35: 159–168. 23380993 10.3233/JAD-122026

[62] Razavi M, Tolea MI, Margrett J, Martin P, Oakland A, et al. (2013) Comparison of 2 Informant Questionnaire Screening Tools for Dementia and Mild Cognitive Impairment: AD8 and IQCODE. Alzheimer Dis Assoc Disord Available: http://journals.lww.com/alzheimerjournal/Abstract/2014/04000/Comparison_of_2_Informant_Questionnaire_Screening.10.aspx. Accessed 9 October 2013. 10.1097/WAD.0000000000000008 PMC3981951 24113559

[63] Ready RE, Ott BR, Grace J (2004) Validity of informant reports about AD and MCl patients' memory. Alzheimer Dis Assoc Disord 18: 11–16. 15195458 10.1097/00002093-200401000-00003

[64] Isella V, Villa L, Russo A, Regazzoni R, Ferrarese C, et al (2006) Discriminative and predictive power of an informant report in mild cognitive impairment. J Neurol Neurosurg Psychiatry 77: 166–171. 16421116 10.1136/jnnp.2005.069765 PMC2077604

[65] Van der Elst W, Van Boxtel MPJ, Van Breukelen GJP, Jolles J (2008) A large-scale cross-sectional and longitudinal study into the ecological validity of neuropsychological test measures in neurologically intact people. Arch Clin Neuropsychol 23: 787–800. 18930628 10.1016/j.acn.2008.09.002

